# A Member of the *14-3-3* Gene Family in *Brachypodium distachyon, BdGF14d*, Confers Salt Tolerance in Transgenic Tobacco Plants

**DOI:** 10.3389/fpls.2017.00340

**Published:** 2017-03-13

**Authors:** Yuan He, Yang Zhang, Lihong Chen, Chunlai Wu, Qingchen Luo, Fan Zhang, Qiuhui Wei, Kexiu Li, Junli Chang, Guangxiao Yang, Guangyuan He

**Affiliations:** The Genetic Engineering International Cooperation Base of Chinese Ministry of Science and Technology, Key Laboratory of Molecular Biophysics of Chinese Ministry of Education, College of Life Science and Technology, Huazhong University of Science and TechnologyWuhan, China

**Keywords:** *Bd14-3-3s*, *BdGF14d*, salt tolerance, ABA signaling, ROS-scavenging system, *B. distachyon*

## Abstract

Plant 14-3-3 proteins are involved in diverse biological processes, but for the model monocotyledonous species, *Brachypodium distachyon*, their roles in abiotic stress tolerance are not well understood. In this study, a total of eight *Bd14-3-3* genes were identified from *B. distachyon* and these were designated respectively as *BdGF14a–BdGF14g*. The qRT-PCR analyses of 3-month-old plants of *B. distachyon* showed that these genes were all expressed in the stems, leaves, and spikelets. By contrast, most of the plants had relatively lower transcriptional levels in their roots, except for the *BdGF14g* gene. The different expression profiles of the *Bd14-3-3s* under various stress treatments, and the diverse interaction patterns between Bd14-3-3s and BdAREB/ABFs, suggested that these gene products probably had a range of functions in the stress responses. The NaCl-induced *Bd14-3-3* gene, *BdGF14d*, was selected for overexpression in tobacco. BdGF14d was found to be localized throughout the cell and it conferred enhanced tolerance to salt in the transgenic plants. Lowered contents of malondialdehyde, H_2_O_2_, and Na^+^, and lower relative electronic conductance (Rec%), yet greater activities of catalase and peroxidase, were observed in the overexpressing plants. Higher photosynthetic rate, transpiration rate, stomatal conductance, and water use efficiency were measured in the transgenic lines. Following abscisic acid (ABA) or NaCl treatment, stomatal aperture in leaves of the *BdGF14d*-overexpression plants was significantly lower than in leaves of the wild type (WT) controls. The stress-related marker genes involved in the ABA signaling pathway, the reactive oxygen species (ROS)-scavenging system, and the ion transporters were all up-regulated in the *BdGF14d*-overexpressing plants as compared with WT. Taken together, these results demonstrate that the *Bd14-3-3* genes play important roles in abiotic stress tolerance. The ABA signaling pathway, the ROS-scavenging system, and ion transporters were all involved in enhancing the tolerance to salt stress in the *BdGF14d*-overexpression plants.

## Introduction

Many processes in the life cycles of plant require specific signal transduction to achieve vital biological functions. A common way to transfer such information is *via* the interaction between proteins, as modified by reversible phosphorylation (Ferl, 2004; Chevalier et al., 2009). However, since reversible phosphorylation of a protein alone is insufficient to complete the interaction directly, 14-3-3s, as crucial adaptor proteins are of great importance in plants because they bind phosphorylated peptides in the regulation of a diversity of processes (Denison et al., 2011), notably primary metabolism (Comparot et al., 2003; Diaz et al., 2011) and light (Sullivan et al., 2009; Taoka et al., 2011) and hormone signaling pathways (Ishida et al., 2004; Schoonheim et al., 2007; Gokirmak et al., 2010; Wang H. et al., 2011). In recent years, an increasing number of studies have shown that 14-3-3s also play important roles in how plants respond to a variety of biotic (Li et al., 2014; Teper et al., 2014) and abiotic (Chen et al., 2006; Sun et al., 2014; Zhou et al., 2014; He et al., 2015) stresses *via* the binding of the phosphorylated target proteins (Obsil and Obsilova, 2011).

The 14-3-3 proteins occur as homo- or hetero-dimers: each subunit forms a highly conserved amphipathic groove consisting of little more than nine antiparallel α-helices for ligand binding, as indicated by the crystal structure solved from tobacco (Wurtele et al., 2003; Ferl, 2004). The structural characteristics of these 14-3-3 proteins equip them for the regulation of various environmental signaling pathways, such as those related to drought, high salinity, and extreme temperatures.

Many recent studies have reported that 14-3-3s are implicated in abiotic responses as determined by an expression assay (Aksamit et al., 2005; Chen et al., 2006; Yao et al., 2007; Denison et al., 2011), while other studies have testified to their importance through investigations of transgenic plants. Cotton (*Gossypium hirsutum*) and *Arabidopsis thaliana* plants overexpressing the *14-3-3* gene *AtGF14λ* (Yan et al., 2004) and *A. thaliana* plants overexpressing the *14-3-3* gene *AtGRF9* (He et al., 2015) each showed improved drought tolerance. On the other hand, a *14-3-3λ* and *14-3-3κ* double mutant showed enhanced salt resistance (Zhou et al., 2014). Potato (*Solanum tuberosum*) plants overexpressing *14-3-3* genes showed higher antioxidant activity than wild type (WT) plants; the converse result was found for transgenic potato plants in which the expression of 14-3-3 proteins was repressed (Lukaszewicz et al., 2002). In the case of *Brachypodium distachyon*, which is emerging as a monocotyledonous plant model, the roles of 14-3-3 proteins in stress responses are much less well understood (Draper et al., 2001).

In our study, a total of eight *14-3-3* genes in *B. distachyon* were identified and their cDNAs were cloned. Here, we present results on the expression profiles, and on the interactions between Bd14-3-3 proteins and BdAREB/ABF transcriptional factors (TFs) in *B. distachyon*, and we also show that transgenic tobacco (*Nicotiana tabacum*) plants overexpressing *BdGF14d* are tolerant to salt stress.

## Materials and Methods

### Identification and Cloning of the *Bd14-3-3* Gene Family from *B. distachyon*

For reference, a total of 13 complete amino acid sequences of *Arabidopsis* 14-3-3s were downloaded from TAIR^1^. The databases of Phytozome v11.0^2^ and the plantGDB^3^ were both used to extensively search for Bd14-3-3s; all the putative 14-3-3 sequences were submitted to Pfam^4^ to validate their 14-3-3 conserved domains, and then they were uploaded to NCBI^5^ to perform BLAST analyses. All the accession numbers of the obtained *Bd14-3-3* genes are listed in Supplementary Table S1 and all the genes were amplified using gene-specific primers (Supplementary Table S2). The exon–intron gene structures were analyzed on BdGDB^6^. Sequence alignments among the Bd14-3-3 family and *Arabidopsis* and rice plants were undertaken using Clustal W. The results were edited by GeneDoc and the phylogenetic tree was constructed with the MEGA6 software tool using the neighbor-joining method (Chen et al., 2012). The promoter sequences were downloaded from PLAZA^7^ and the *cis*-elements in the promoters were analyzed on PlantCARE^8^.

### Analysis of the Gene Expression Profiles by qRT-PCR

Seeds of the *B. distachyon* inbred line, Bd21 were germinated in distilled water under a photoperiod of 16-h light/8-h darkness. For RNA extraction, leaf samples of 2-week-old seedlings treated with 200 mM NaCl, 20% PEG6000, 10 mM H_2_O_2_, and 100 μM abscisic acid (ABA) were collected at 0, 1, 3, 6, 12, and 24 h after treatments. Different organs of 3-month-old plants of *B. distachyon* were also sampled, namely the roots, stems, leaves, and spikelets. Total RNA was extracted using a plant tissue total RNA extraction kit (Zomanbio, Beijing, China), the first cDNA chain was synthesized using the PrimeScript^TM^ RT reagent Kit with a gDNA Eraser (Takara, Japan). The qRT-PCR analysis was performed using the SuperReal PreMix Plus (SYBR Green, Tiangen, Beijing, China) on a qRT-PCR machine (Bio-Rad, Hercules, CA, USA). The primer sequences used for the expression analysis are given in Supplementary Table S3.

### Yeast Two-Hybrid Assay

The yeast two-hybrid assay was performed according to reported methods (Sun et al., 2015). Eight *BdAREB/ABFs* were isolated during the identification of the *Bd14-3-3s*. The *Bd14-3-3s* and *BdAREB/ABFs* were incorporated into pGBKT7 and pGADT7 eukaryotic expression vectors. The primers used for the cloning of the *BdAREB/ABF*s and the vector constructions are shown in Supplementary Tables S4 and S5.

### Generation of Transgenic Lines, and Salt-Stress Tolerance Assays

The complete cDNA sequence of *BdGF14d* was introduced into the eukaryotic expression vector pBI121 as driven by the *CaMV* 35S promoter. The transformations of tobacco by the *Agrobacterium tumefaciens* strain EHA105 containing recombinant plasmids or empty vectors, respectively, were performed as described elsewhere (Huang et al., 2015). Three overexpressed lines were screened on 100 mg/L kanamycin and confirmed by qRT-PCR tests using gene-specific primers were selected for salt-tolerance assay (**Figure 5A**). Seven-day-old seedlings of the WT and vector control (VC) plants and of the overexpressing lines were transferred from plates to a 1/2 MS (Murashige–Skoog) solid medium containing 150 or 200 mM NaCl. Their root lengths were measured after 2-weeks of treatments. Two-week-old seedlings of the five lines cultured on plates were planted in soil and grown under normal conditions for an additional 3 weeks. A 500 mM NaCl solution was used to treat the seedlings every 2 days for 20 days, after which plant physiological indices were measured. Measurement of relative electronic conductance (Rec%) was performed as described previously (Yang et al., 2017). The contents of H_2_O_2_ and malondialdehyde (MDA) and the activity of peroxidase (POD) and catalase (CAT) were determined using spectrophotometric methods and commercially available detection kits ^9^. Measurements of Na^+^ and K^+^ contents were carried out as described for *Arabidopsis* (Sun et al., 2015). Photosynthetic rate, transpiration rate, and stomatal conductance were measured using the LI-6400 portable photosynthesis system (LI-COR Inc., Lincoln, NE, USA), and the water use efficiency (WUE) was calculated as described (Msanne et al., 2011).

### Stomatal Aperture Measurements

The stomatal aperture in leaves was measured as described preciously (Grondin et al., 2015). Six-week-old tobacco leaves of the transgenic lines and the WT plants were sampled and treated with 200 mM NaCl and 50 μM ABA.

### qRT-PCR Analyses of Related Marker Genes

Two-week-old transgenic plants and WT lines cultured on plates were transferred to MS solid medium containing 200 mM NaCl and grown for 7 days, after which entire seedlings were sampled for RNA extraction. The primers used for the qRT-PCR of the specific marker genes involved in salt tolerance are listed in Supplementary Table S6 (Deng et al., 2013a,b).

### Subcellular Localization Analysis of BdGF14d

The subcellular localization of BdGF14d protein was investigated as described for tobacco plants (Hu et al., 2015). The recombinant plasmid 35S::*BdGF14d*-*GFP* was used to perform the assay.

### Statistical Analysis

All data were analyzed by reported methods (Wang et al., 2015).

## Results

### Cloning of the *14-3-3* Genes from *B. distachyon*

**FIGURE 1:**
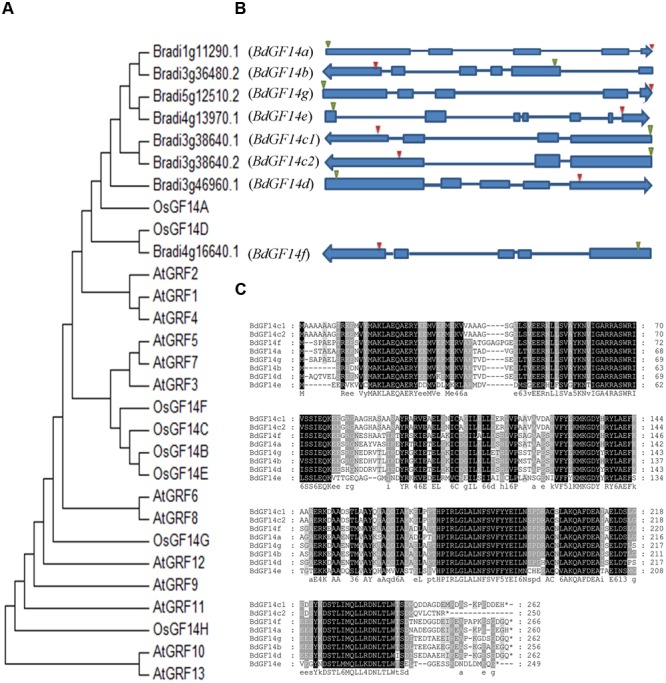
**Phylogenetic and gene structure analyses of the *Bd14-3-3s*. (A)** Phylogenetic tree of 14-3-3s from *B. distachyon*, rice, and *Arabidopsis* plants constructed using MAGA6. **(B)** Gene structures of *Bd14-3-3s*. Solid blue bars and strings represent the exons and introns, respectively; green and red triangles represent the translation starts and stops, respectively. Arrows indicate transcriptional directions on the chromosomes. **(C)** Amino acid sequence alignments of the Bd14-3-3 family.

A total of eight *Bd14-3-3* genes were identified from *B. distachyon* through the BLAST searches and the Pfam analyses; they were designated as *BdGF14a, BdGF14b, BdGF14c1, BdGF14c2, BdGF14d, BdGF14e, BdGF14f*, and *BdGF14g*, respectively, based on the nomenclature in rice (Chen et al., 2006) and their chromosomal location, which was inconsistent with previous report (Cao et al., 2016) that we identified an alternative splicing in *BdGF14c*. BdGF14c1 and BdGF14c2 had the same sequences of 1–244 amino acid residues, but the last 18 amino acid residues for BdGF14c1 and the last six amino acid residues for BdGF14c2 were different, due to the alternative splicing patterns of the *BdGF14c* gene (**Figure 1**). All the *Bd14-3-3s* cDNAs were cloned using gene-specific primers (Supplementary Table S2) from *B. distachyon* and a multiple full-length amino acid sequence alignment, a phylogenetic analysis against *Arabidopsis* and rice, and an exon–intron structural annotation were performed. The results indicated that the Bd14-3-3 family members in *B. distachyon* were highly conserved during evolution (**Figure 1A**), in accordance with their highly similar amino acid alignment (**Figure 1C**). The exon–intron structure analysis showed that most of the *Bd14-3-3s* had three to five exons and two to four introns, apart from *BdGF14e*, which was composed of seven exons and six introns (**Figure 1B**). According to their different gene structures, *BdGF14a, BdGF14b, BdGF14c1, BdGF14c2, BdGF14d, BdGF14f*, and *BdGF14g* were classified into the non-𝜀 group, whereas *BdGF14e* was classified into the 𝜀 group, based on the taxonomy in *Arabidopsis* (DeLille et al., 2001). To explore the putative *cis*-elements involved in the abiotic stress response in promoters, 1 kb sequences upstream of the *Bd14-3-3s* CDS was downloaded and the ensuing abiotic stress-responsive *cis*-elements were analyzed. It was found that all the *Bd14-3-3* genes except *BdGF14d* had one or two ABA-responsive elements (ABREs); the *BdGF14b, BdGF14c1, BdGF14c2*, and *BdGF14f* contained a low-temperature responsive element (LTR); and the *BdGF14a, BdGF14b, BdGF14c1, BdGF14c2*, and *BdGF14g* harbored a heat-stress responsive element (HSE) (Supplementary Table S1). Taken together, these results indicated, that the *Bd14-3-3* genes could participate in these abiotic stresses.

### Interactions between Bd14-3-3s and BdAREB/ABF TFs Proteins

**FIGURE 2:**
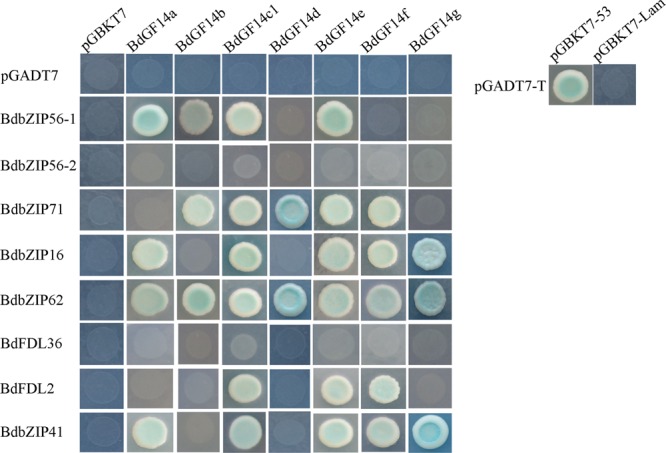
**Yeast two-hybrid analyses between the Bd14-3-3s and the BdAREB/ABF TFs.** The yeast strain AH109, transferred along with the described plasmid combinations, was spotted onto Trp-/Leu-/His-/Ade-medium containing x-α-gal. The *Bd14-3-3s* were cloned into pGBKT7, and the *BdAREB/ABFs* were cloned into pGADT7 vectors. Transformants containing pGADT7-*T* and pGBKT7-*53* vs. pGBKT7-*Lam* represent the positive vs. negative controls, respectively.

Recent work has shown that the interactions between basic leucine zipper (bZIP) and the 14-3-3 proteins can influence numerous plant biological processes, such as the ABA-mediated signaling involved in abiotic stress responses. The *AREB/ABF* genes involved in ABA signaling that belong to subgroup A of *bZIPs* were cloned to verify the interactions between BdAREB/ABF TFs and the Bd14-3-3 proteins. The results demonstrated that all the Bd14-3-3s showed interactions with two to six BdAREB/ABF TFs (**Figure 2**), except for BdGF14c2 which was self-activated (data not shown). Among these, all the Bd14-3-3 family members were able to interact with BdbZIP62, but they interacted with neither BdbZIP56-2 nor BdFDL36. BdGF14c1 and BdGF14e exhibited the same interaction mode, in that both of them interacted with the same six BdAREB/ABF TFs. BdGF14d interacted with only two BdAREB/ABF TF members. The other Bd14-3-3 proteins, BdGF14a, BdGF14b, BdGF14f, and BdGF14g, showed interactions with four, three, five, and three BdAREB/ABF TFs, respectively. As signal transduction in plants always occurs *via* protein–protein interactions, these results suggested that the Bd14-3-3 proteins likely participated in the ABA-mediated signaling pathway by interacting with the BdAREB/ABF TF members.

### Expression Profiles of *Bd14-3-3s* in Different Plant Organs

**FIGURE 3:**
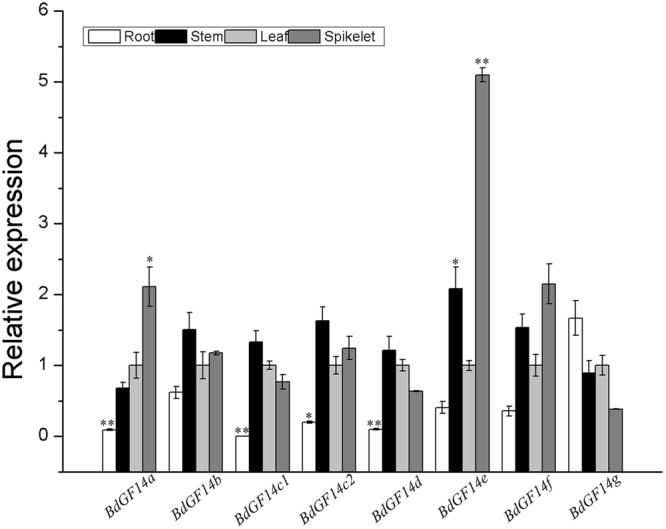
**Expression of the *Bd14-3-3s* in different plant organs.** Roots, stems, leaves, and spikelets of 3-month-old plants of *B. distachyon* were sampled to extract RNA for expression analysis. The *y*-axis represents the relative expression of the genes. All bars are means (±SE) calculated from three replicates; asterisks denote significant differences between leaves and the other organs (^∗^*P* < 0.05; ^∗∗^*P* < 0.01).

To investigate the biological function of *Bd14-3-3s*, the expression profiles of these *Bd14-3-3s* in the roots, stems, leaves, and spikelets of 3-month-old plants of *B. distachyon* were investigated. The results indicated that most members of this gene family except *BdGF14g* had relatively lower expression levels in the roots than in the leaves (**Figure 3**). Besides this differences, all the *Bd14-3-3* genes exhibited global expression in the stems, leaves and spikelets. Notably, *BdGF14a* and *BdGF14e* showed significantly higher expression levels in the spikelets, pointing to the putative functional involvement of these two genes in seed development.

### Expression Profiles of *Bd14-3-3s* in Response to Abiotic Stresses

**FIGURE 4:**
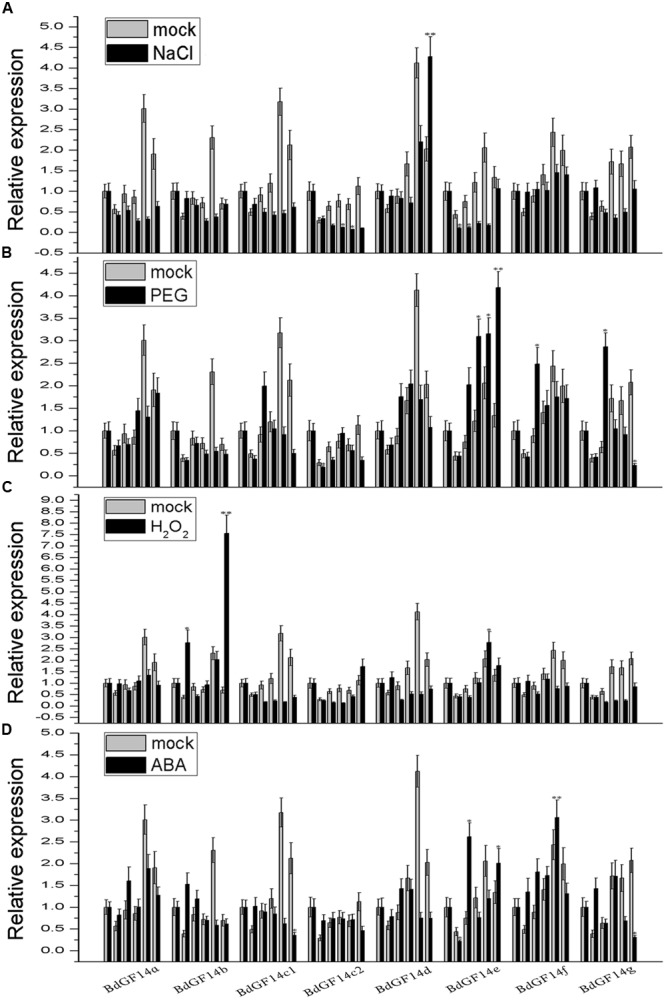
**Expression profiles of *Bd14-3-3s* under applied stress treatments.** Leaves of *B. distachyon* were collected at 0, 1, 3, 6, 12, and 24 h after being treated with 200 mM NaCl **(A)**, 20% PEG **(B)**, 10 mM H_2_O_2_ **(C)**, and 100 μM ABA **(D)**, respectively, and RNA was extract for expression analyses. Mock indicates the control (no treatment applied). Bars are means (±SE), all the experiments were repeated three times. Asterisks denote significant differences between 0 h and different time points (^∗^*P* < 0.05; ^∗∗^*P* < 0.01).

Plant 14-3-3 proteins play important roles in response to various abiotic stresses (Aksamit et al., 2005; Sun et al., 2014; He et al., 2015). Therefore, qRT-PCR analyses were performed to determine the expression patterns of the *14-3-3* gene family under conditions of abiotic stress PEG6000, NaCl, and the signaling molecules ABA and H_2_O_2_. Considering that the fluctuation of the *Bd14-3-3s* expression was influenced by photoperiod in the control, the results revealed that only *BdGF14d* showed distinct up-regulation in response to the NaCl treatment, whereas the other genes exhibited different degrees of down-regulation (**Figure 4A**). Under the PEG treatment, *BdGF14e* was up-regulated; however, *BdGF14f* and *BdGF14g* were up-regulated at 3 h after 20% PEG6000 was added (**Figure 4B**). In response to H_2_O_2_, *BdGF14b* showed evidence of up-regulation, whereas *BdGF14c1* and *BdGF14d* exhibited down-regulation; the other genes demonstrated moderate variation (**Figure 4C**). Under the ABA treatment, *BdGF14e* and *BdGF14f* showed evidence of up-regulation, and *BdGF14b* and *BdGF14g* were up-regulated rapidly at 1 h after treatment but were then down-regulated at 12 h; the other genes exhibited no significant variation in response to ABA (**Figure 4D**). The expression patterns of the *Bd14-3-3* gene family suggested that different *14-3-3* isoforms played specific roles in the response to diverse abiotic stresses, and that, correspondingly, the respective 14-3-3 proteins might participate in the signaling transduction network of this response.

### Overexpression of *BdGF14d* Enhances Salt Tolerance in Transgenic Tobacco Plants

**FIGURE 5:**
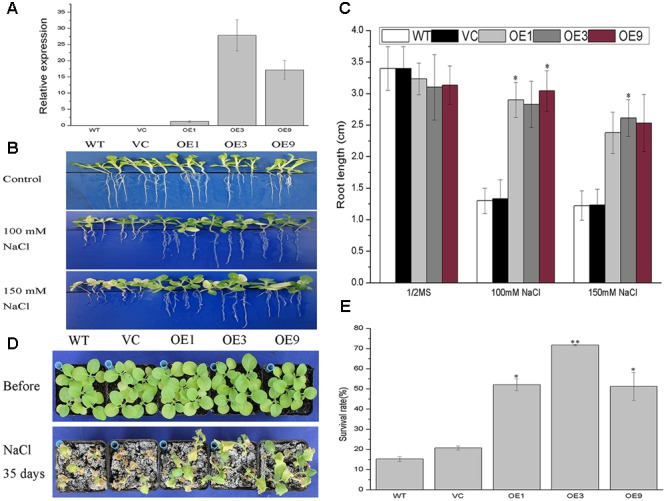
**Improved tolerance to salt in the *BdGF14d*-overexpression plants. (A)** The relative expression levels of *BdGF14d* in transgenic tobacco plants. The *Ntubiquitin* was used as internal reference. **(B)** Seven-day-old seedlings of WT, VC, and transgenic lines germinated on MS were transferred to 1/2 MS containing 100 mM and 150 mM NaCl to grow for a further 2 weeks. **(C)** The root lengths were measured and statistically analyzed. **(D)** Two-week-old seedlings growing on MS medium were planted in soil to grow under normal condition for 3 weeks and 500 mM NaCl solution was used to treat the seedlings for a further 35 days. **(E)** The respective survival rates were calculated. All the experiments were repeated three times. Bars are means (±SE); asterisks denote significant differences between WT and the transgenic lines (^∗^*P* < 0.05; ^∗∗^*P* < 0.01).

Although functional redundancy is common in a multigene family such as *14-3-3s, BdGF14d* was the only gene in the *Bd14-3-3s* family to respond to NaCl, based on the expression profiles. Hence, *BdGF14d* was selected for a salt tolerance study by overexpressing it in transgenic tobacco plants. Three transgenic lines screened on 100 mg/L kanamycin and confirmed by qRT-PCR analyses using gene-specific primers were selected for this salt tolerance assay (**Figure 5A**). To evaluate the performance of the *BdGF14d*-overexpression seedlings under extensive salt stress, the 7-day-old seedlings of the WT, VC, and transgenic lines that germinated on a MS medium were transferred to 1/2 MS medium containing 100 and 150 mM NaCl to grow for a further 2 weeks. The *BdGF14d*-overexpression lines developed distinctly longer roots than WT under both levels of the NaCl salt treatment 100 and 150 mM (**Figure 5B**); yet they showed no apparent difference from WT and VC lines under normal conditions (**Figure 5C**). The 3-week-old seedlings of the three *BdGF14d*-overexpression and WT, VC lines were treated with 500 mM NaCl for 35 days in soil and significantly higher survival rates (52.1, 71.7, and 51.3%, respectively) than WT (15.2%) and VC (20.7%) were observed. Moreover, the leaves of transgenic seedlings maintained their growing status, whereas those of the WT and VC plants had already wilted or died (**Figure 5D**), yet there was no apparent difference between WT, VC and transgenic lines under normal conditions (**Figure 5E**).

**FIGURE 6:**
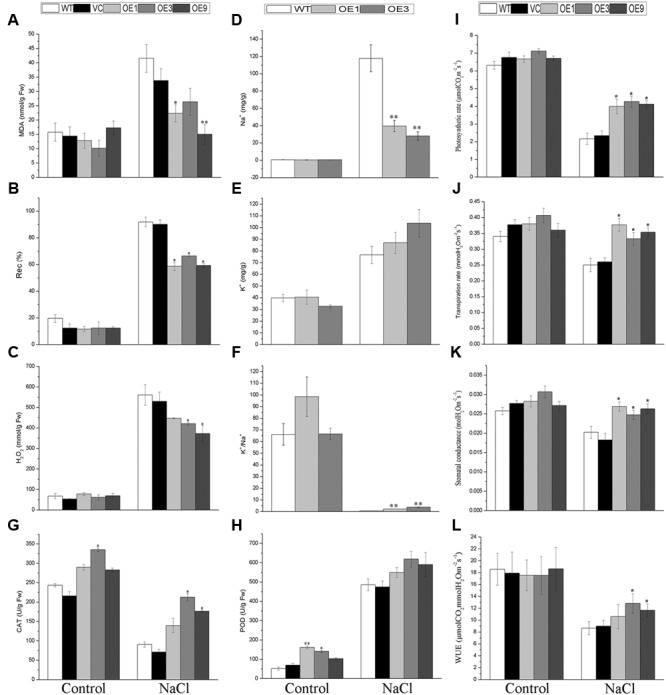
**Plant physiological indices of transgenic lines and WT.** Two-week-old WT and transgenic lines geminated on MS were planted in soil to grow for 3 weeks; all seedlings were treated with a 500 mM NaCl solution every 2 weeks for a further 20 days. Leaves of the five lines with or without salt applied were sampled for measurements of MDA **(A)**, Rec% **(B)**, H_2_O_2_ **(C)**, CAT **(G)**, POD **(H)**, photosynthetic rate **(I)**, transpiration rate **(J)**, stomatal conductance **(K)**, WUE **(L)**, and for their contents of Na^+^ **(D)** and K^+^ **(E)**. The ratio of K^+^/Na^+^ was also calculated **(F)**. Bars are means (±SE); and asterisks denote significant differences between WT and the transgenic lines (^∗^*P* < 0.05; ^∗∗^*P* < 0.01). All the experiments were repeated three times.

Salt stress will promote the accumulation of reactive oxygen species (ROS), which leads to oxidative damage (Hossain and Dietz, 2016). To investigate the salt tolerance mechanism in the *BdGF14d*-overexpression seedlings in physiological terms, the related indices of MDA, Rec%, and H_2_O_2_ these reflect membrane and intracellular oxidative damage and the antioxidant enzyme system of CAT and POD were determined in transgenic lines and control plants after NaCl treatment or under normal growing conditions. The results showed that the *BdGF14d*-overexpression plants had obviously lower contents of MDA and H_2_O_2_ and lower Rec% after the NaCl treatment (**Figures 6A–C**). In the transgenic lines, the activity of CAT was significantly higher than in WT under the salt stress and normal condition; likewise, POD activity of transgenic lines was clearly higher under normal condition. Interestingly, the POD activity of overexpressing plants showed a slight increase than that of WT after the salt treatment (**Figures 6G,H**). The above results suggested that in the *BdGF14d*-overexpression plants tolerance to salt was enhanced by activating the antioxidant enzymes to mitigate oxidative damage.

To determine the accumulation of Na^+^ and K^+^, these two cations were measured in the leaves of transgenic and WT plants with or without salt stress applied. The results showed that, under salt stress, the transgenic lines accumulated significantly less Na^+^ and possessed distinctly higher K^+^/Na^+^ ratio in leaves than did the WT plants (**Figures 6D,F**). By contrast, the contents of K^+^ showed no significant variation in all plants tested (**Figure 6E**).

To explore the WUE of WT and the overexpressing plants under normal conditions and under NaCl treatment, their photosynthetic rate, transpiration rate, and stomatal conductance were measured. The results indicated that the *BdGF14d*-overexpression plants had a higher photosynthetic rate, transpiration rate, stomatal conductance, and WUE than did the WT plants under salt stress; however, no significant differences were observed between the two plant types under normal condition (**Figures 6I–L**).

### Overexpression of *BdGF14d* Promotes Stomatal Closure under NaCl and ABA Conditions

**FIGURE 7:**
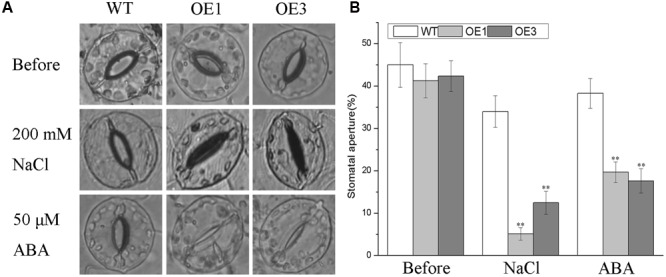
**Analysis of stomatal aperture in transgenic lines and WT plants under the NaCl and ABA treatments.** Detached leaves of WT and transgenic lines were dipped in stomatal opening buffer under light for 6 h. A total of 200 mM NaCl and 50 μM ABA were added to treat the leaves for 1 and 2 h, respectively **(A)** and the stomatal apertures were calculated **(B)**. Bars are means (±SE); asterisks denote significant differences between WT and the transgenic lines (^∗∗^*P* < 0.01).

Salt stress facilitates the accumulation of endogenous ABA, which is a vital regulator of stomatal closure to reduce the amount of Na^+^ transported from roots to shoots and to minimize water loss *via* transpiration (Kim et al., 2010; Campos et al., 2016). The differences in morphology and physiology observed above between the transgenic lines and WT plants under salt stress prompted us to investigate whether the overexpressing *BdGF14d* in tobacco plants affected the stomatal closure in seedlings treated with NaCl or ABA. No apparent differences were observed when the transgenic and WT plants were dipped in a bathing solution without NaCl or ABA in the light. However, there were stark differences in stomatal aperture when detached leaves were treated with either NaCl or ABA (**Figure 7**). Specifically, under either treatment, the degree of stomatal closure in leaves was significantly greater in the *BdGF14d*-overexpression plants than in the WT plants. These results suggested that overexpressing *BdGF14d* accelerated stomatal closure under NaCl stress and that this mechanism to enhance salt tolerance was dependent upon ABA signaling.

### The qRT-PCR Analyses of Marker Genes Involved under Salt Stress

**FIGURE 8:**
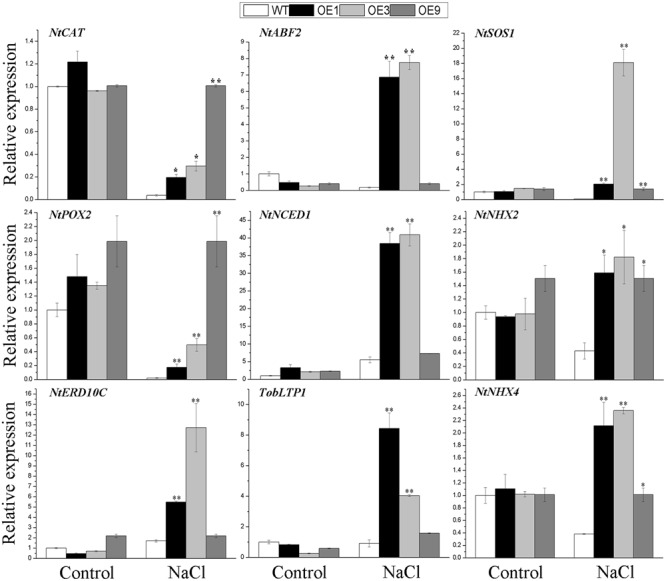
**The qRT-PCR analyses of related marker genes of the transgenic lines and WT under salt stress.** Two-week-old seedlings that germinated on MS medium were transferred to MS containing 200 mM NaCl to grow for 7 days. Entire seedlings of the five lines with or without NaCl applied were sampled to extract RNA for expression analyses. Bars are means (±SE); asterisks denote significant differences between WT and the transgenic lines (^∗^*P* < 0.05; ^∗∗^*P* < 0.01).

To further determine the effects of *BdGF14d* in the overexpressing plants, related marker genes involved in the response to salt stress were amplified by qRT-PCR, with or without the NaCl treatment. A crucial ABA biosynthetic gene *NtNCED1*, a key target of the ABA signaling gene, *NtABF2*, and an ABA-induced gene *TobLTP1* were all significantly up-regulated under salt stress in transgenic lines, relative to WT, albeit no distinct differences were observed without salt applied. The ROS-scavenging genes *NtCAT* and, *NtPOX2*, and the stress defense gene *NtERD10C*, showed obvious up-regulation in the *BdGF14d*-overexpression plants as compared with WT under the salt treatment. The expression levels of the plasma membrane Na^+^/H^+^ antiporter gene *NtSOS1* and of both tonoplast Na^+^/H^+^ antiporters genes, *NtNHX2* and *NtNHX4*, showed evident up-regulation in the overexpressed lines as compared with the WT plants under salt (**Figure 8**). These expression results suggested that BdGF14d appeared to confer salt tolerance to transgenic plants *via* changes in ABA signaling, ROS-scavenging, ion transporters, and cellular protective proteins (such as NtERD10Cs).

### BdGF14d is Localized throughout the Cell

**FIGURE 9:**
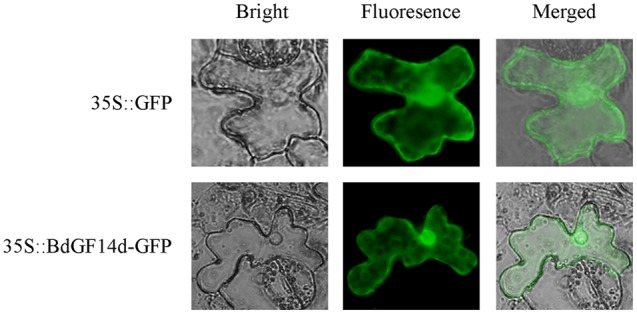
**Subcellular localization of the BdGF14d protein.** *BdGF14d* and *GFP* were inserted into the pBI121 vector, and the recombinant plasmid pBI121-*BdGF14d*-*GFP* and vector control pBI121 were introduced into *A. tumefaciens* for injection into the epidermal cells of tobacco leaves.

To determine the subcellular localization of the BdGF14d protein, the leaf epidermal cells of tobacco plants were injected with *A. tumefaciens* that contained the recombinant plasmid pBI121-*BdGF14d*-*GFP*. The results showed that the BdGF14d was localized throughout the cell (**Figure 9**).

## Discussion

The scaffolding protein 14-3-3 family plays a vital role in signaling networks in eukaryotic cells by binding the phosphorylated target proteins to form dynamic protein complexes, which directly decides the interaction strength of proteins through (de)phosphorylation (Tzivion et al., 2001; Sehnke et al., 2002). An increasing number of studies have reported the importance of 14-3-3 proteins in the tolerance of abiotic stress, but their exact roles in the *B. distachyon* plant species, a newly emerging monocotyledon model, have not yet been explored.

In this study, eight *14-3-3* genes in *B. distachyon* were identified and preliminary functional analyses were successfully carried out. An alternative splicing of *BdGF14c1* containing different exon–intron gene structures was found, this was designated as *BdGF14c2*, which differed from the other seven *14-3-3* genes identified in a previous report (Cao et al., 2016). The promoter *cis*-elements analyses of the eight *Bd14-3-3* genes suggested their potential functions involved in response to abiotic stresses and in ABA signaling. Expression profiles of the *Bd14-3-3s* in the different organs showed relatively lower expression levels in roots but higher levels in the spikelets (**Figure 3**), suggesting that the Bd14-3-3s likely participated in seed development. Further, the expression profiles of the *Bd14-3-3* gene family under abiotic stresses and ABA treatment showed isoform-specific up- or down-regulation (**Figure 4**), thus implying their various roles in the abiotic stress responses of *B. distachyon*. The crucial roles of the 14-3-3 proteins in the light-signaling pathway have been clarified extensively in *Arabidopsis* (Sullivan et al., 2009; Gao et al., 2014) and rice (Taoka et al., 2011), and certain light-responsive elements were identified in promoters of the *Bd14-3-3s* (data not shown). Hence, when these are taken into consideration, up-regulation of the expression levels of the *Bd14-3-3s* following exposure to light was expected under controlled condition. After taking into account the fluctuating expression levels of the *Bd14-3-3* in control plants, the significant up-regulation of *BdGF14d* when plants were treated with NaCl, and likewise that of *BdGF14e, BdGF14f*, and *BdGF14g* with PEG, of *BdGF14b* with H_2_O_2_, and of *BdGF14e* and, *BdGF14f* with ABA, together indicates that Bd14-3-3s seem to be involved in diverse abiotic stress and ABA responses.

Previous studies have demonstrated that the 14-3-3 proteins can exert critical effects in plant response to abiotic stresses (Yan et al., 2004; Sun et al., 2014; He et al., 2015). In our study, the *BdGF14d*-overexpression tobacco plants had significantly higher tolerance to salt stress than did WT plants. This result was confirmed by the several physiological index measurements and verified further by the qRT-PCR analyses of the related marker genes in the transgenic lines and WT plants. ABA clearly plays a pivotal role in the diverse adaptive stress responses to unfavorable environmental stimuli including high salinity, drought and extreme temperatures as an important plant hormone (Fujita et al., 2011; de Boer et al., 2013). In *Arabidopsis*, the phosphorylated ABF3 combines with 14-3-3 to stabilize its conformation and thereby facilitate ABA signaling (Sirichandra et al., 2010), and in barley plants the 14-3-3s responded to ABA and controlled ABA action (Schoonheim et al., 2007).

To better explore the putative role of ABA in the improved salt tolerance of transgenic plants, we carried out (1) a yeast two-hybrid assay between the Bd14-3-3s and BdAREB/ABF TFs in *B. distachyon*, (2) a stomatal aperture test under the ABA treatment, and (3) qRT-PCR analyses of related marker genes known to be involved in ABA signaling. The interactions between BdGF14d and an ABA-insensitive 5 (ABI5)-like protein, BdbZIP62, and a transcription factor responsible for ABA regulation 1 (TRAB1)-like protein BdbZIP71 involved in ABA signaling in *B. distachyon*, were both confirmed by the yeast two-hybrid assay, thus suggesting that BdGF14d was indeed involved in the ABA signaling pathway (**Figure 2**). The adaptive responses to abiotic stresses as mediated by ABA include stomatal closure and the transcription of a number of related genes involved in the stress tolerance process (Choi et al., 2000; An et al., 2008; Wang R.S. et al., 2011; Geilfus et al., 2015; Campos et al., 2016). The 14-3-3 proteins seem to participate in ABA-mediated stomatal closure (Cotelle and Leonhardt, 2016): there was less stomatal aperture in leaves of the *BdGF14d*-overexpression plants than in those of WT under the ABA treatment (**Figure 7**); this implicates *BdGF14d* in ABA-mediated stomatal closure. The higher expression levels of three ABA signaling related genes *NtNCED1* that controls the biosynthesis of ABA, *NtABF2* that facilitates various ABA-mediated signal transductions, and *TobLTP1* that responds to ABA (Zhang et al., 2005; Deng et al., 2013b; Hu et al., 2015) found in the *BdGF14d*-overexpression plants (vs. WT; **Figure 8**), suggests that BdGF14d promoted ABA signaling transduction. From the above set of results, we infer that the BdGF14d probably enhanced tolerance to salt in the transgenic plants *via* ABA signaling.

Excess ROS accumulation induced by diverse abiotic stresses generates oxidative damage in the plasma membrane and intracellularly, and so plants have evolved effective antioxidant enzyme systems for ROS detoxification (Liu and He, 2016; Rao and Chaitanya, 2016). The relatively higher activity of the antioxidant enzymes confer upon maize an improved tolerance to environmental stimuli (Stepien and Klobus, 2005). Maintaining a strong antioxidant defense system is of great importance to plants for enhanced tolerance to high salt condition (Hu et al., 2012; Hossain and Dietz, 2016). In the present study, there was relatively higher activity of CAT and POD, which both belong to the antioxidant enzyme, in the *BdGF14d*-overexpression lines than in the WT plants (**Figures 6G,H**). Similarly, *NtCAT* and *NtPOX2* also exhibited higher expression levels in the transgenic lines when under salt stress (**Figure 8**). These results revealed that the *BdGF14d*-overexpression plants had improved salt tolerance that was linked to an enhanced ROS-scavenging system.

High salinity inhibits the growth and reproduction of plants *via* excessive osmotic stress and ion damage (Deinlein et al., 2014; Lv et al., 2014). However, plants have evolved physiological mechanisms, such as the inhibition of stomatal openings to limit the transportation of Na^+^ from roots to leaves by transpiration, as well as the reduction of Na^+^ accumulation in the leaf cytoplasm by excreting Na^+^ out and compartmentalizing excessive Na^+^ into vacuoles, so that they can continue to function while under salt stress (Zhu, 2003; Zhang and Shi, 2013). Increased activity of the plasma membrane Na^+^/H^+^ antiporter, SOS1, and of the vacuolar Na^+^/H^+^ antiporters, NHXs, confers plants salt tolerance to plants by transporting excess Na^+^ from the cytoplasm out of the cell and into vacuoles, respectively, to reduce the toxic levels of Na^+^ (Li et al., 2009; Barragan et al., 2012; Zhang and Shi, 2013; Roy et al., 2014). Further, an increased abundance of 14-3-3s enhances the activity of PM H^+^-ATPase, thus generating an H^+^ electrochemical gradient that provides the driving force for SOS1 to mitigate abiotic stresses (Bunney et al., 2002; Kerkeb et al., 2002; Fuglsang et al., 2007; Pallucca et al., 2014). To resist salt stress in particular, the transcriptional levels of the vacuolar Na^+^/H^+^ exchangers *NHXs* are up-regulated by ABA to facilitate stomatal closure and intracellular ionic homeostasis (Apse et al., 1999; Zhu, 2003; Barragan et al., 2012). In the present study, the higher expression levels of *NtSOS1, NtNHX2*, and *NtNHX4* (**Figure 8**) and the lower accumulation of Na^+^ (**Figure 6D**) in the *BdGF14d*-overexpressing plant leaves (vs. WT), together, indicate that the regulation of ion transporters by BdGF14d was involved in the improvement of salt stress tolerance.

The photosynthetic rate and WUE in the *BdGF14d*-overexpression plants were higher than in WT plants under salt stress; but interestingly, the transpiration rate and stomatal conductance were also higher in the transgenic lines (**Figures 6I–L**). This outcome was probably due to the complicated regulatory mechanism of the whole plant and the dynamic physiological changes across the different growth stages of seedlings.

## Conclusion

Eight *Bd14-3-3* genes were identified and cloned from *B. distachyon* plants. The different expression profiles of the *Bd14-3-3* gene family under the different stress treatments tested, coupled to the diverse interaction patterns between Bd14-3-3s and BdAREB/ABF TFs, suggest various roles for these genes in abiotic stress responses involving ABA. Overexpression of *BdGF14d*, a NaCl-induced member of the *Bd14-3-3* gene family, enhanced tolerance to salt stress in the transgenic tobacco plants. The ROS-scavenging system and ion transporters contributed to salt resistance, while ABA signaling was involved in the enhanced salt tolerance of the *BdGF14d*-overexpression plants. Together, the findings provide valuable information for elucidating the roles and complex mechanisms of plant 14-3-3s in response to diverse abiotic stresses.

## Author Contributions

GH, GY, JC, and YH designed the experiments and wrote the paper. YH performed all experiments and analyzed the data. YZ and LC helped to identify the genes. YZ helped to conduct the experiments. CW, QL, FZ, QW, and KL participated in partial experiments. All authors read and approved the manuscript.

## Conflict of Interest Statement

The authors declare that the research was conducted in the absence of any commercial or financial relationships that could be construed as a potential conflict of interest.

## Footnotes

1 http://www.arabidopsis.org

2 https://phytozome.jgi.doe.gov/pz/portal.html

3 http://www.plantgdb.org

4 http://pfam.xfam.org

5 https://www.ncbi.nlm.nih.gov

6 http://www.plantgdb.org/BdGDB

7 http://bioinformatics.psb.ugent.be/plaza

8 http://bioinformatics.psb.ugent.be/webtools/plantcare/html

9 www.njjcbio.com

## Abbreviations

ABA: abscisic acid
ABI5: abscisic acid insensitive 5
AREB/ABF: ABA responsive element binding factor
bZIP: basic leucine zipper
*CaMV*: *Cauliflower mosaic virus*
CAT: catalase
CDS: coding sequence
ERD10C: early responsive to dehydration 10C
GA: gibberellin
GFP: green fluorescent protein
HSE: heat stress responsive element
LTR: low-temperature responsive element
MDA: malondialdehyde
MS: Murashige–Skoog
NCED1: 9-*cis*-epoxycarotenoid dioxygenase 1
NHX: Na^+^/H^+^ antiporter
OE: overexpression
POD: peroxidase
qRT-PCR: quantitative real-time polymerase chain reaction
Rec%: relative electronic conductance
ROS: reactive oxygen species
TFs: transcription factors
TobLTP1: tobacco lipid transfer protein 1
TRAB1: transcription factor responsible for ABA regulation 1
UTR: untranslated region
VC: vector control
WT: wild type
WUE: water use efficiency
