# Supplementary material for: A Member of the 14-3-3 Gene Family in Brachypodium distachyon, BdGF14d, Confers Salt Tolerance in Transgenic Tobacco Plants
Source: Front Plant Sci. 2017 Mar 13;8:340. doi: 10.3389/fpls.2017.00340 (PMC5346558; doi:10.3389/fpls.2017.00340)
Supplement: Supplementary file 1 [file Table_1.DOCX]

Table S1. Analyses of the *cis*-elements involved in abiotic stresses of the *Bd14-3-3s*

| No. | Subgroup | Gene Name | Accession Number | Chr. | *Cis*-elements of promotor | | |
| --- | --- | --- | --- | --- | --- | --- | --- |
|  |  |  |  |  | ABRE | LTR | HSE |
|  |  |  |  |  |  |  |  |
| 1 | non-ε group | *BdGF14a* | KU933262 | 1 | 1 | 0 | 1 |
| 2 |  | *BdGF14b* | KU933264 | 3 | 1 | 1 | 1 |
| 3 |  | *BdGF14c1* | KU933259 | 3 | 1 | 1 | 1 |
| 4 |  | *BdGF14c2* | KU933260 | 3 | 1 | 1 | 1 |
| 5 |  | *BdGF14d* | KU933265 | 3 | 0 | 0 | 0 |
| 6 |  | *BdGF14e* | KU933266 | 4 | 2 | 0 | 0 |
| 7 |  | *BdGF14f* | KU933261 | 5 | 1 | 1 | 0 |
| 8 | ε group | *BdGF14g* | KU933263 | 4 | 1 | 0 | 1 |
