# Supplementary material for: A Member of the 14-3-3 Gene Family in Brachypodium distachyon, BdGF14d, Confers Salt Tolerance in Transgenic Tobacco Plants
Source: Front Plant Sci. 2017 Mar 13;8:340. doi: 10.3389/fpls.2017.00340 (PMC5346558; doi:10.3389/fpls.2017.00340)
Supplement: Supplementary file 2 [file Table_2.DOCX]

Table S2. Primers used for constructions of pBI121-*Bd14-3-3s*

| Gene Name | Forward/reverse primers |
| --- | --- |
| *BdGF14a* | 5’-GCTCTAGAATGTCTACTGCTGAGGCAACC-3’ |
|  | 5’-CGGGATCCGTGACCCTCTCCTTCAGGC-3’ |
| *BdGF14b* | 5’-GCTCTAGAATGTCGCGGGAGGACAATGT-3’ |
|  | 5’-CGGGATCCCTGGCCCTCGCCAGCT-3’ |
| *BdGF14c1* | 5’-GCTCTAGAATGGCTGCAGCGGCGGCGG-3’ |
|  | 5’-CGGGATCCGTGCTCATCATCCTCAGGC-3’ |
| *BdGF14c2* | 5’-GCTCTAGAATGGCTGCAGCGGCGGCGG-3’ |
|  | 5’-CGGGATCCCCGGTTAGTACACAATACCTGC-3’ |
| *BdGF14d* | 5’-GCTCTAGAATGGCACAGACTGTGGAGCT-3’ |
|  | 5’-CGGGATCCCTGTCCGTCTCCAGATTCTC-3’ |
| *BdGF14e* | 5’-GCTCTAGAATGGAGGAGAGGGTGAAGGT-3’ |
|  | 5’-CGGGATCCTCCCTGCTCCATATCGAGG-3’ |
| *BdGF14f* | 5’-GCTCTAGAATGTCGCCGGCGGAGCCG-3’ |
|  | 5’-CGGGATCCCTGTCCATCTCCAGATTCTTTTG-3’ |
| *BdGF14g* | 5’-GCTCTAGAATGTCGGCACCTGCGGAGCT-3’ |
|  | 5’-CGGGATCCCTGCCCATCACCAGAGTCA-3’ |

|  |
| --- |
