# Supplementary material for: A Member of the 14-3-3 Gene Family in Brachypodium distachyon, BdGF14d, Confers Salt Tolerance in Transgenic Tobacco Plants
Source: Front Plant Sci. 2017 Mar 13;8:340. doi: 10.3389/fpls.2017.00340 (PMC5346558; doi:10.3389/fpls.2017.00340)
Supplement: Supplementary file 4 [file Table_4.DOCX]

Table S4. Primers used for constructions of pGADT7-*BdAREB/ABFs*

| Gene Name | Forward/reverse primers |
| --- | --- |
| *BdbZIP56.1* | 5’-GGAATTCCATATG ATGGATCTGGGGGACGGC-3’ |
|  | 5’-CGGAATTCTTACCAGGGACCTGTGAGTGTTCTC-3’ |
| *BdbZIP56.2* | 5’-GGAATTCCATATGATGGATCTGGGGGACGGC-3’ |
|  | 5’-CGGAATTCTTAGCGCAGCCTCTTGCAACCAC-3’ |
| *BdbZIP71* | 5’-GGAATTCCATATGATGCTGACGGAGGACAGGTG-3’ |
|  | 5’-CGGGATCCC TTACCAAGGACCAGTCAATGTTCT-3’ |
| *BdbZIP16* | 5’-CGGAATTC ATGGAGATGCCGGGAGGGAG-3’ |
|  | 5’-CGGGATCCC CTACCATGGACCGGTCAGCGT-3’ |
| *BdbZIP62* | 5’-CGGAATTC ATGGATTTTCCGGGAGGGAG-3’ |
|  | 5’-CGGGATCCC TCACGGCCCTGTCAGTGTCC-3’ |
| *BdFDL36* | 5’-GGAATTCCATATG ATGAGCTCCCAAGGTGG-3’ |
|  | 5’-CGGGATCCC TCACTGTTTCCTAGCTCTTGAACG-3’ |
| *BdFDL2* | 5’-GGAATTCCATATG ATGATTCAGGCAATGTCGTCG-3’ |
|  | 5’-CGGAATTCTCAAACAGGGGAAGAGCTTGTTC-3’ |
| *BdbZIP41* | 5’-GCTCTAGAATGGCGTCACAACCCGGGC-3’ |
|  | 5’-CGGGATCCGAAAGCCGCTGAGCATGTTCTC-3’ |
