# Supplementary material for: A Member of the 14-3-3 Gene Family in Brachypodium distachyon, BdGF14d, Confers Salt Tolerance in Transgenic Tobacco Plants
Source: Front Plant Sci. 2017 Mar 13;8:340. doi: 10.3389/fpls.2017.00340 (PMC5346558; doi:10.3389/fpls.2017.00340)
Supplement: Supplementary file 5 [file Table_5.DOCX]

Table S5. Primers used for constructions of pGBKT7-*Bd14-3-3s*

| Gene Name | Forward/reverse primers |
| --- | --- |
| *BdGF14a* | 5’-GGAATTCCATATGATGTCTACTGCTGAGGCAACC-3’ |
|  | 5’-CGGGATCCCTTAGTGACCCTCTCCTTCAGGC-3’ |
| *BdGF14b* | 5’-CGGAATTCATGTCGCGGGAGGACAATGT-3’ |
|  | 5’-CGGGATCCCTTACTGGCCCTCGCCAGCT-3’ |
| *BdGF14c1* | 5’-CGGAATTCATGGCTGCAGCGGCGGCGG-3’ |
|  | 5’-CGGGATCCCCTAGTGCTCATCATCCTCAGGC-3’ |
| *BdGF14c2* | 5’-CGGAATTCATGGCTGCAGCGGCGGCGG-3’ |
|  | 5’-CGGGATCCCTTACCGGTTAGTACACAATACCTGC-3’ |
| *BdGF14d* | 5’-CGGAATTCATGGCACAGACTGTGGAGCT-3’ |
|  | 5’-CGGGATCCCTTACTGTCCGTCTCCAGATTCTC-3’ |
| *BdGF14e* | 5’-CGGAATTCATGGAGGAGAGGGTGAAGGT-3’ |
|  | 5’-CGGGATCCCTTATCCCTGCTCCATATCGAGG-3’ |
| *BdGF14f* | 5’-CGGAATTCATGTCGCCGGCGGAGCCG-3’ |
|  | 5’-CGGGATCCCTTACTGTCCATCTCCAGATTCTTTTG-3’ |
| *BdGF14g* | 5’-AACTGCAGATGTCGGCACCTGCGGAGCT-3’ |
|  | 5’-GGATCCCGATGTCGGCACCTGCGGAGCT-3’ |
