# Supplementary material for: A Member of the 14-3-3 Gene Family in Brachypodium distachyon, BdGF14d, Confers Salt Tolerance in Transgenic Tobacco Plants
Source: Front Plant Sci. 2017 Mar 13;8:340. doi: 10.3389/fpls.2017.00340 (PMC5346558; doi:10.3389/fpls.2017.00340)
Supplement: Supplementary file 6 [file Table_6.DOCX]

Table S6. Primers used for qRT-RCR analyses of related marker genes

| Gene Name | Forward/reverse primers |
| --- | --- |
| *NtNCED1* | 5’-AAGAATGGCTCCGCAAGTTA-3’ |
|  | 5’-GCCTAGCAATTCCAGAGTGG-3’ |
| *NtABF2* | 5’-GCAGCCATCTATCTATTC-3’ |
|  | 5’-GCAACTCATCCATATTCA-3’ |
| *TobLTP1* | 5’-GGTTTTGTGCATGGTGGTGG-3’ |
|  | 5’-CTTAGAGCAGTCTGTGGAGG-3’ |
| *NtCAT* | 5’-AGGTACCGCTCATTCACACC-3’ |
|  | 5’-AAGCAAGCTTTTGACCCAGA-3’ |
| *NtPOX2* | 5’-CTTGGAACACGACGTTCCTT-3’ |
|  | 5’-TCGCTATCGCCATTCTTTCT-3’ |
| *NtERD10C* | 5’-AACGTGGAGGCTACAGATCG-3’ |
|  | 5’-GTTCCTCTTGGGCATGAGTT-3’ |
| *NtNHX2* | 5’-ACTCATCCCCATTGGTCCG-3’ |
|  | 5’-AAGGAGTTCCACAAAAGCACGA-3’ |
| *NtNHX4* | 5’-CAAGAACTTCCGCACCCAC-3’ |
|  | 5’-GCAGTATCAAACGCAGAGGACC-3’ |
| *NtSOS1* | 5’-CAAATGTTATCCCCCGAAAGC-3’ |
|  | 5’-CGGAGAACCTGAGGAAATGTGA-3’ |
| *Nt**ubiquitin* | 5’-AAAGAGTCAACCCGTCACCT-3’ |
|  | 5’-ACATCACGACCACAACCAGA-3’ |
